# Optimal Resource Allocation for Loss-Tolerant Multicast Video Streaming

**DOI:** 10.3390/e25071045

**Published:** 2023-07-11

**Authors:** Sadaf ul Zuhra, Karl-Ludwig Besser, Prasanna Chaporkar, Abhay Karandikar, H. Vincent Poor

**Affiliations:** 1 Department of Electrical and Computer Engineering, Princeton University, Princeton, NJ 08544, USA; karl.besser@princeton.edu (K.-L.B.); poor@princeton.edu (H.V.P.); 2 Department of Electrical Engineering, Indian Institute of Technology Bombay, Mumbai 400076, India; chaporkar@ee.iitb.ac.in (P.C.); karandi@ee.iitb.ac.in (A.K.)

**Keywords:** multicast, video streaming, loss tolerance, MBMS, resource allocation

## Abstract

In video streaming applications, especially during live streaming events, video traffic can account for a significant portion of the network traffic and can lead to severe network congestion. For such applications, multicast provides an efficient means to deliver the same content to a large number of users simultaneously. However, in multicast, if the base station transmits content at rates higher than what can be decoded by users with the worst channels, these users will experience outages. This makes the multicast system’s performance dependent on the weakest users in the system. Interestingly, video streams can tolerate some packet loss without a significant degradation in the quality experienced by the users. This property can be leveraged to improve the multicast system’s performance by reducing the dependence of the multicast transmissions on the weakest users. In this work, we design a loss-tolerant video multicasting system that allows for some controlled packet loss while satisfying the quality requirements of the users. In particular, we solve the resource allocation problem in a multimedia broadcast multicast services (MBMS) system by transforming it into the problem of stabilizing a virtual queuing system. We propose two loss-optimal policies and demonstrate their effectiveness using numerical examples with realistic traffic patterns from real video streams. It is shown that the proposed policies are able to keep the loss encountered by every user below its tolerable loss. The proposed policies are also able to achieve a significantly lower peak SNR degradation than the existing schemes.

## 1. Introduction

The popularity of video streaming platforms such as Netflix and YouTube has led to a fundamental shift in the way that video content is consumed online. Users increasingly prefer to stream content on the go over cellular wireless networks. As a result, during live video streaming events (such as the Super Bowl, Facebook, YouTube, Instagram live sessions, etc.), the same video content is transmitted to thousands of users over orthogonal spectral resources. This massive influx of video traffic consumes a substantial fraction of the limited amount of spectrum available for use by cellular systems, leading to severe network congestion and degraded quality of service. For such services, multicast transmission provides an excellent solution [1,2] that can serve users over shared spectral resources while also improving the quality of service.

A major bottleneck in multicast transmissions is that, in order to serve all user equipments (UEs) in a multicast group, data cannot be transmitted at a rate greater than what can be decoded by the UE with the weakest channel in the group. As a result, UEs with good channel conditions are constantly forced to settle for lower rates despite their high channel quality indicator (CQI) values, leading to user dissatisfaction and low system throughput. This work proposes a novel method to overcome these issues by exploiting the loss-tolerant nature of video streams. It has been shown that video streams can tolerate as much as 40% packet loss [3] without significantly impacting the quality observed by the end users. For instance, for an H.264/AVC encoded video, decoders such as FFmpeg and JM can conceal as much as 39% packet loss with no deterioration in the quality of video observed by the users [3]. This can be leveraged to build video-specific resource allocation policies that can significantly reduce the bandwidth consumption of video streams.

A compressed video stream is made up of a group of pictures (GoP). A GoP comprises a series of intra-coded (I), predicted (P) and bidirectional predicted (B) frames. I frames are self-contained and do not require other frames to be decoded. P frames are dependent on their preceding I frames to be correctly decoded, and B frames are dependent on both preceding and following I and/or P frames to be correctly decoded. Although the actual number of I, P and B frames in a GoP depends upon the size of the GoP used, the number of B frames is at least twice the number of I and P frames combined [4].

It is difficult to estimate the impact of the loss of I and P frames on the video quality [5]. However, since B frames encode differential information with respect to the past and future I and P frames, their loss has the least impact on the quality of the video. Therefore, in this work, we assume that the base station uses lossless allocation policies [2] to allocate sufficient resources for the lossless transmission of I and P frames, and the lossy transmission proposed here impacts only the B frames of a video stream.

### 1.1. Related Literature

In this section, we present the relevant state of the art for the problem addressed in this work. The relevant literature can be broadly considered under the following three categories. (a) The study of problems related to optimal resource allocation in wireless multicast transmission. These include multicast transmission for video streaming, as well as other forms of data. (b) The study of the problem of joint grouping and resource allocation in wireless multicast transmission. The grouping problem refers to the problem of creating multicast groups of UEs, which could be based on the content requested by the UEs, the channel quality of UEs or, in the case of multi-layer video streaming, the number of enhancement layers that a UE can receive. (c) The study of optimal multicast streaming strategies specifically for multi-layer video transmission.

The most important literature from each of these categories is summarized in the following subsections.

#### 1.1.1. Resource Allocation

A resource allocation algorithm for live video streaming that allocates resources based on the channel quality and priority of UEs is proposed in [6]. The proposed policy makes use of streaming statistics to reserve resources for UEs that have priority in the system. In [7], the authors propose a frequency domain packet scheduler (FDPS) for multimedia broadcast multicast services (MBMS) that maximizes the minimum rate achievable by UEs in a physical resource block (PRB). It uses a conservative approach that only minimizes the damage caused by the worst PRB assignment. Video delivery simultaneously using WiFi unicast and 4G multicast has been proposed in [8], with the aim of minimizing the load on 4G while maximizing the quality of video received by the user. Methods enabling multi-connectivity for multicast video streaming have been proposed in [9]. In [10], a scheduling scheme for MBMS broadcast is proposed that is focused on reducing the average latency of packets in the system. The proposed scheme starts transmission in unicast mode and gradually moves to broadcast as the number of UEs increases. In [11], the authors deal with efficient broadcasting in LTE using MBMS. The resource allocation algorithm proposed in [11] uses a water filling form of proportional fair scheduling [12,13]. In [14], an SDN-based video streaming architecture is proposed for the IP multicasting of advanced video-coded live streams. The proposed architecture aims at minimizing the bandwidth usage and cost of transcoding for live streaming. More recently, various learning techniques have also been employed to solve the problem of resource allocation in multicast streaming. In [15], deep reinforcement learning is used for resource allocation in multicast TV services.

#### 1.1.2. Joint Grouping and Resource Allocation for Multicast Transmission

The problem of grouping and resource allocation for lossless multicast streaming has been studied in [1,2]. The objective of resource allocation in [1,2] is to satisfy all the multicast UEs while minimizing the number of PRBs used in doing so. In [16], the authors propose a fair and optimal resource allocation policy for MBMS. It is assumed that the video content is simultaneously available through unicast and MBMS and the primary problem seeks to jointly optimize the grouping of UEs and the allocation of resources to unicast and MBMS services. In [17], the problem of joint power allocation and subgrouping is addressed in a non-orthogonal multiple access (NOMA)-based multi-layer multicast streaming system. The algorithms proposed in [17] are aimed at achieving the minimum target rate and proportional fairness for the base layers of the video streams that carry the most essential content. The problem of joint user grouping, version selection and resource allocation for multicast streaming in a cloud RAN framework has been studied in [18].

#### 1.1.3. Multi-Layer Video Transmission

Resource allocation for MBMS operation on-demand has been studied in [19]. The authors consider quality of experience (QoE) metrics such as user engagement, instead of quality of service (QoS) metrics such as throughput, as the utility functions to be maximized by the resource allocation schemes. All the video streams are assumed to be encoded using scalable video coding (SVC). In [20], convex optimization is used to obtain an optimal solution for the multicasting of dynamic adaptive streaming over HTTP (DASH) [21] and for SVC streaming of content over LTE. The problem optimizes the modulation and coding schemes and the forward error correction code rates used while allocating resources. An adaptive resource assignment scheme for scalable video multicast has been proposed in [22], with the objective of maximizing the long-term quality of experience of the system.

In [23], the authors use a pricing-based scheme for the allocation of resources to multicast groups streaming SVC video content. Users are divided into three multicast groups based on the price that they pay. The UEs that pay the most receive the maximum number of enhancement layers. In [24], the authors investigate the use of random network linear coding (RNLC) to improve the performance of multicast services. They use two different forms of RNLC for the multicasting of H.264/SVC videos in a generic cellular system. The authors in [25] deal with optimizing the delivery of network-coded SVC content using MBMS. They make use of unequal error protection to ensure the reliability of multi-layer video transmission. A resource allocation model that provides better coverage than conventional multi-rate transmission is also proposed in [26].

### 1.2. Contributions

Existing approaches do not leverage the unique loss-tolerant nature of video streams to optimize resource allocation in multicast video streaming. This work exploits this property to design efficient resource allocation policies for video multicasting. A loss-tolerant mechanism for video streaming is proposed that allows for controlled packet losses without significantly impacting the quality of service. Each UE has a certain tolerance for loss, which could be a function of several factors, such as the type of video being streamed, the type of subscription (for instance, a costlier subscription would imply lower loss tolerance), the device being used to stream the video or the channel quality experienced by the UE.

Moreover, most of the existing wireless multicast literature assumes the rate achievable by a UE to be the same across all PRBs. This assumption significantly simplifies the resource allocation problem. Without the channel variability over PRBs, all PRBs are equivalent for a multicast group/UE and the problem of resource allocation is simplified to only determining the number of PRBs to be allocated to a multicast group. This work takes into account the fact that, due to fast fading, the CQI of a UE may also vary for different PRBs within a sub-frame. Therefore, while determining the allocation of PRBs to groups, the identity of the PRBs to be allocated also needs to be specified.

The main contributions of this paper are summarized below. Since multicast services in fifth-generation (5G) communications are termed MBMS, the terms MBMS and multicast services will be used interchangeably through the rest of this paper.

- A loss-tolerant mechanism for multicast video streaming is proposed that exploits the loss-tolerant nature of videos to improve the system performance and utilize the available bandwidth more efficiently. The proposed mechanism allows for controlled packet losses while satisfying the quality of service requirements of the users.
- The problem of resource allocation in loss-tolerant MBMS systems is converted to the problem of stabilizing a fictitious virtual queuing system. It is proven that stabilizing the constructed virtual token queues is equivalent to satisfying the loss requirements of the users (Section 4).
- Two loss-optimal policies are proposed for the allocation of resources in loss-tolerant MBMS systems, namely loss optimal resource allocation (LORA) and priority LORA (p-LORA). An algorithm for the efficient polynomial time implementation of these policies is also provided (Section 5). These policies do not require any statistical information about the channel states of users. Channel states can vary arbitrarily and can also be correlated across users. The proposed policies are optimal in the sense that they can satisfy the loss requirements of all the UEs whenever any other policy, including offline policies with complete information of channel states of users, can do so.
- The performance of the proposed policies is evaluated using extensive simulations. Since these policies are designed for video streaming, traces from actual videos [4,5] are used to simulate realistic video traffic patterns (Section 6).

Unlike the multicast streaming mechanisms in the existing literature (Section 1.1.1 and Section 1.1.2), the proposed loss-tolerant streaming mechanism allows a larger number of users to be served within the same spectral resources and avoids network congestion during peak traffic hours. Contrary to conventional multicast transmission [2,16], loss-tolerant streaming reduces the dependence of a multicast group on the UE with the worst channel quality, as the resource allocation policy is no longer constrained to serving every UE in every sub-frame. Therefore, the transmission rates in some sub-frames may be higher than what can be decoded by the weakest UEs, resulting in higher system throughput and better user satisfaction.

Although we do not consider multi-layered video transmission in this work, the proposed policies can also be extended to these applications by considering each enhancement layer as a separate stream. In this case, the loss tolerances would also be a function of the enhancement layer being transmitted.

#### Notation

Vectors are written in boldface letters, e.g., $B={\left(B_{1},\ldots ,B_{N}\right)}^{T}$. The set of integers up to *n* is denoted as [n]={1,2,…,n}. As a shorthand, we use ${\left[x\right]}^{+}=\max\left\{x,0\right\}$. The probability and expectation operators are denoted by Pr and E, respectively. An overview of the most commonly used variables’ notation can be found in Table 1.

## 2. System Model

Consider a 5G multicast system with *L* different video streams. There are *M* UEs in the system, each subscribed to one of the *L* video streams. UEs subscribed to video stream i∈{1,2,…,L} form multicast group $G_{i}$ and the number of UEs in $G_{i}$ is denoted by $K_{i}$. The index of the group that UE *k* belongs to is denoted by i(k), i.e., if UE *k* belongs to the group $G_{j}$, then i(k)=j. Thus, [M] and [L] denote the set of UEs and the set of multicast groups, respectively. In each sub-frame, there are N≥L PRBs that can be assigned to the groups. Since these PRBs are typically shared with other types of data transmission in the system, each multicast group $G_{i}$ is allocated at most one PRB in each sub-frame.

For each of the *L* video streams, a data packet arrives at the beginning of each sub-frame and is transmitted in the same sub-frame. The size of the arriving packet for group $G_{i}$, along with the length of a sub-frame, determines the rate $R_{i}$ (in bits/second) at which the data needs to be transmitted to its subscribers. Therefore, whenever a PRB is allocated to multicast group $G_{i}$, data is transmitted in this PRB at the corresponding rate $R_{i}$.

In each sub-frame t∈N, the resource allocation policy Γ decides which PRB is allocated to which group. This allocation in sub-frame *t* is denoted in form of the allocation vector $B^{\Gamma }\left[t\right]$ of length *L* given by

(1) $$B^{\Gamma }\left[t\right]={\left(B_{1}^{\Gamma }\left[t\right],B_{2}^{\Gamma }\left[t\right],\ldots ,B_{L}^{\Gamma }\left[t\right]\right)}^{T},$$

where $B_{i}^{\Gamma }\left[t\right]=j\in \left\{0,1,\ldots ,N\right\}$ describes that PRB *j* is allotted to $G_{i}$ in sub-frame *t*. However, if $B_{i}^{\Gamma }\left[t\right]=0$, it means that group $G_{i}$ is not scheduled for reception in this sub-frame. The policy Γ in sub-frame *t* is completely defined by the value of $B^{\Gamma }\left[t\right]$.

The channel states of the UEs vary across time and frequency. As a result, the channel experienced by a UE varies from one sub-frame to another and also across the PRBs within a sub-frame. There is a certain maximum rate $r_{kj}\left[t\right]$ that UE *k* can successfully decode in PRB *j* of sub-frame *t* [27]. This rate is a function of the CQI experienced by the UE in this PRB. Since data is transmitted to group $G_{i}$ at rate $R_{i}$, a UE may not receive the MBMS content successfully, even after a PRB has been assigned to its multicast group. A UE is said to have been *served* in a sub-frame if and only if the UE successfully receives data in this sub-frame. Therefore, even if the group of the UE is *scheduled* for reception in a sub-frame, the UE itself may or may not be *served* in this sub-frame. We distinguish between these two terms as follows.

- A UE is *scheduled* in a sub-frame, if a PRB is allocated to its group in this sub-frame. More precisely, UE $k\in G_{i}$ is scheduled in sub-frame *t* under policy Γ if $B_{i}^{\Gamma }\left[t\right]\neq 0$.
- A UE is *served* in a sub-frame, if it has been scheduled in that sub-frame and is able to successfully receive the transmitted packet. More precisely, UE $k\in G_{i}$ is served in sub-frame *t* under policy Γ if $B_{i}^{\Gamma }\left[t\right]=j\neq 0$ and $R_{i}\leq r_{kj}\left[t\right]$.

Recall that for each video stream *i*, a packet arrives at the beginning of each sub-frame. Therefore, if a UE is not served in a sub-frame, it experiences a packet loss. We denote the loss encountered by UE *k* under policy Γ in sub-frame *t* by $\ell_{k}^{\Gamma }\left[t\right]$. More precisely, for UE k∈[M], the loss $\ell_{k}^{\Gamma }\left[t\right]$ under policy Γ in sub-frame *t* is given by

(2) $$\ell_{k}^{\Gamma }\left[t\right]=\left\{\begin{array}{cc} 0 & \mathrm{if}\;B_{i(k)}^{\Gamma }\left[t\right]\neq 0\;\mathrm{and}\;R_{i(k)}\leq r_{kj}\left[t\right],\\ 1 & \mathrm{otherwise}. \end{array}\right.$$

All UEs in the system can tolerate some degree of packet loss. This loss tolerance may differ from UE to UE depending upon the channel conditions experienced and the video resolution chosen by them. A higher resolution would typically imply lower loss tolerance and vice versa. For all k∈[M], we denote by ${\tilde{\ell }}_{k}$ the fractional loss that can be tolerated by UE *k*. The loss tolerance vector for the system is given by

(3) $$\tilde{\ell }={\left({\tilde{\ell }}_{1},\ldots ,{\tilde{\ell }}_{M}\right)}^{T}.$$

Within this framework, the objective of the resource allocation problem is to allocate PRBs to the multicast groups such that the average loss encountered by every UE *k* is below its tolerable loss ${\tilde{\ell }}_{k}$.

**Example** **1.** *In order to illustrate the described model, we consider a simple example of M=5 UEs, each subscribing to one of L=3 streams. In particular, users *1*, *2*, and *4* form group $G_{1}$, while users *3* and *5* are groups $G_{2}$ and $G_{3}$, respectively. In each sub-frame, there are N=2 PRBs available. For this example, we assume that the policy *Γ* assigns PRB *1* to group $G_{3}$ and PRB *2* to group $G_{1}$ in sub-frame t, i.e., we have $B^{\Gamma }\left[t\right]={\left(2,0,1\right)}^{T}$.*

*The frame that needs to be transmitted for video stream *1* has a size of 100 kbit, while the packet of stream *3* is 80 kbit. Since the length of a sub-frame in 5G-NR is 1 *ms*, the required data rates for groups *1* and *3* are $R_{1}=100\;\mathrm{Mbit}/s$ and $R_{3}=80\;\mathrm{Mbit}/s$, respectively. Assuming that the users can decode packages up to rates $r_{1}=120\;\mathrm{Mbit}/s$, $r_{2}=90\;\mathrm{Mbit}/s$, $r_{4}=100\;\mathrm{Mbit}/s$, and $r_{5}=90\;\mathrm{Mbit}/s$, we find that users *2* and *3* experience losses due to not being able to decode and not being scheduled, respectively. The loss vector for this example is therefore given according to (2) as $\ell^{\Gamma }\left[t\right]={\left(0,1,1,0,0\right)}^{T}$.*

## 3. Problem Definition

The main problem considered in this work is to find an efficient resource allocation policy that satisfies the different loss tolerances of the UEs. For the exact formulation of the problem, we require the following definitions.

**Definition** **1** (Feasible resource allocation)**.** *Resource allocation under policy *Γ* in sub-frame t is said to be* feasible *if, at most, one PRB is assigned to each multicast group and no two groups are assigned the same PRB. More precisely, a feasible allocation vector $B^{\Gamma }\left[t\right]$ is such that, for all $\left(i,i^{\prime }\right)\in {\left[L\right]}^{2}$ with $B_{i}^{\Gamma }\left[t\right],B_{i^{\prime }}^{\Gamma }\left[t\right]\neq 0$, it holds that $B_{i}^{\Gamma }\left[t\right]\neq B_{i^{\prime }}^{\Gamma }\left[t\right]$.*

**Definition** **2** (Feasible resource allocation policy)**.** *A feasible resource allocation policy *Γ* is a policy that chooses a feasible allocation vector in each sub-frame.*

A resource allocation policy can make use of the knowledge of the current channel states of the UEs, the allocation information of the previous sub-frames, the loss tolerance of the UEs, and the losses encountered by the UEs in the previous sub-frames to make allocation decisions in a sub-frame. It could also be an off-line policy that has prior knowledge of the channel conditions of all sub-frames in advance. However, we will show in the following sections that this prior knowledge does not improve the performance and that the proposed policies achieve optimal performance without requiring any knowledge of future channel conditions.

**Definition** **3** (Average packet loss)**.** *The average packet loss encountered by UE k under resource allocation policy *Γ* is the packet loss per unit time given by*

$$\bar{\ell_{k}^{\Gamma }}=\underset{T\to \infty }{lim sup}\frac{1}{T}\sum_{t=1}^{T}\ell_{k}^{\Gamma }\left[t\right],$$

*with $\ell_{k}^{\Gamma }\left[t\right]$ in (2).*

The vector of the average packet losses of all UEs is given by

(4) $$\bar{\ell^{\Gamma }}={\left(\bar{\ell_{1}^{\Gamma }},\ldots ,\bar{\ell_{M}^{\Gamma }}\right)}^{T}.$$

The feasible region of a resource allocation policy and that of the system can now be defined as follows.

**Definition** **4** (Feasible region of a policy)**.** *The feasible region of a resource allocation policy *Γ*, denoted by $L^{\Gamma }$, is the set of all loss tolerance vectors $\tilde{\ell }$ that can be satisfied by *Γ*, i.e.,*

(5) $$L^{\Gamma }=\left\{\tilde{\ell }:\tilde{\ell }>\bar{\ell^{\Gamma }}\;a.s.\right\}\;,$$

*where $\tilde{\ell }$ is defined in (3) and $\bar{\ell^{\Gamma }}$ in (4).*

**Definition** **5** (Feasible region of the system)**.** *The feasible region of the system is the set of loss vectors $L={⋃}_{\Gamma }L^{\Gamma }$ where the union is over all feasible policies *Γ*.*

Using the above definitions, the optimal resource allocation policy can now be defined as follows.

**Definition** **6** (Optimal resource allocation policy)**.** *The optimal resource allocation policy $\Gamma^{★}$ is a policy for which the feasible region is given by $L^{\Gamma^{★}}={⋃}_{\Gamma }L^{\Gamma }$.*

**Problem** **Statement.** Based on the above definitions, the main objective of this work is to determine the optimal resource allocation policy $\Gamma^{★}$ from Definition 6.

**Remark** **1.** *While the focus of this work is on video streaming, it should be emphasized that the considered problem and our proposed resource allocation framework are not uniquely applicable to video streaming, but more generally to any loss-tolerant multicast transmission.*

## 4. A Virtual Queuing System for Multicast Resource Allocation

**Figure 1:**
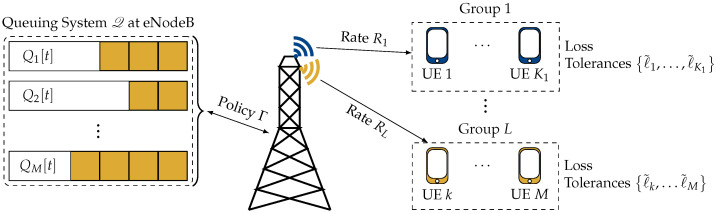
Considered MBMS system in which *M* users subscribe to one of *L* video streams. Each UE *k* has an average loss tolerance ${\tilde{\ell }}_{k}$. The base station maintains a virtual queuing system Q, which keeps track of the packet losses for the individual users. Based on the state of Q, the resource allocation policy Γ assigns a PRB to each group i∈{1,2,…,L}, which corresponds to transmitting the data at rate $R_{i}$.

In this section, we show that the considered problem can be solved by converting it into the problem of stabilizing a queuing system. In particular, we construct a virtual queuing system Q, which consists of a token queue for each UE. The term *token* is used to refer to the virtual entities that make up the queues, and the tokens are used to keep track of the losses of the individual UEs. The basic idea is that tokens in each queue arrive at a rate proportional to the loss tolerance of the corresponding user. For users with stricter reliability constraints, i.e., lower loss tolerances, more tokens arrive on average. Whenever a UE is served, a token is removed from the corresponding queue. Thus, the length of the queue of a user is an indicator of how much loss a user has encountered. The state of this queuing system is completely described by the lengths of these virtual queues. An overview of the system is depicted in Figure 1.

For all k∈[M], the arrival process for the token queue of UE *k* is denoted by ${\left\{\lambda_{k}\left[t\right]\right\}}_{t\geq 1}$. The variable $\lambda_{k}\left[t\right]$ is a binary random variable indicating the arrival of a token to the queue of UE *k* in sub-frame *t* and has the expected value ${\bar{\lambda }}_{k}=1-{\tilde{\ell }}_{k}$. Therefore, if the virtual queue *k* with this expected arrival rate ${\bar{\lambda }}_{k}$ is stabilized, we ensure that UE *k* is served in at least $1-{\tilde{\ell }}_{k}$ of the sub-frames. Arrivals across sub-frames are assumed to be independent and identically distributed. Across users, the arrival processes are assumed to be independent. The system arrival rate vector is denoted by $\lambda ={\left({\bar{\lambda }}_{1},\ldots ,{\bar{\lambda }}_{M}\right)}^{T}$.

Denote by $\mu_{k}^{\Gamma }\left[t\right]$ the binary random variable that indicates whether or not UE *k* has been served in sub-frame *t* under policy Γ, i.e., $\mu_{k}^{\Gamma }\left[t\right]$ is given by

(6) $$\mu_{k}^{\Gamma }\left[t\right]=\left\{\begin{array}{cc} 1, & \mathrm{if}\;B_{i(k)}^{\Gamma }\left[t\right]=j\neq 0\;\mathrm{and}\;R_{i(k)}\leq r_{kj}\left[t\right],\\ 0, & \mathrm{otherwise}. \end{array}\right.$$

Let $Q_{k}^{\Gamma }\left[t\right]$ denote the length of queue *k* at the beginning of sub-frame *t* under policy Γ. For all k∈[M], the queue length $Q_{k}^{\Gamma }\left[t\right]$ evolves according to the following:

(7) $$Q_{k}^{\Gamma }\left[t+1\right]={\left[Q_{k}^{\Gamma }\left[t\right]+\lambda_{k}\left[t\right]-\mu_{k}^{\Gamma }\left[t\right]\right]}^{+}.$$

As mentioned above, we will show in the following that stabilizing the virtual queuing system Q provides a solution to the originally considered resource allocation problem. For this, we introduce the following definitions.

**Definition** **7** (Stability of Q)**.** *The constructed queuing system Q is said to be stable under a feasible resource allocation policy *Γ* if it holds that $\sup_{t}E\left[Q_{k}^{\Gamma }\left[t\right]\right]<\infty$, for all k∈[M].*

A resource allocation policy that stabilizes Q is called a *stable resource allocation policy*. The stability region of a stable resource allocation policy and the queuing system Q are defined as follows.

**Definition** **8** (Stability region of Γ)**.** *The stability region $S_{\Gamma }$ of a stable resource allocation policy *Γ* is the set of arrival rate vectors for which the system is stable under* Γ.

**Definition** **9** (Stability region of Q)**.** *The stability region S of the queuing system Q is the union of the stability regions of all stable resource allocation policies, $S={⋃}_{\Gamma }S_{\Gamma }$, where the union is over all stable *Γ*.*

**Definition** **10** (Throughput optimality)**.** *A resource allocation policy *Γ* is said to be throughput-optimal [28] if *Γ* can stabilize the queuing system Q provided that the queuing system is stabilizable.*

Since the eNodeB (eNB) knows both the loss requirements and the channel states of the UEs, it has all the information needed to maintain the virtual queuing system. In the following, we establish the relationship between the stability region of the constructed queuing system and the feasible region of the optimal resource allocation policy $\Gamma^{★}$ from Definition 6.

### Feasible Region of the Optimal Resource Allocation Policy

In this section, we prove that stabilizing the constructed virtual queuing system Q is equivalent to meeting the loss requirements of all UEs in the system. This establishes the equivalence between the stability region of Q and the feasible region of the optimal resource allocation policy $\Gamma^{★}$ defined in Definition 6. The consequence of establishing this equivalence is that designing a resource allocation policy that stabilizes Q is equivalent to solving the originally considered problem.

Let $B=\{B_{1},\ldots ,B_{\left|B\right|}\}$ be the set of all feasible allocation vectors of the form in (1). The cardinality of B is given by

|B|=N+1LL!+∑k=0LLkNL−k(L−k)!,

where *N* is the number of PRBs in a sub-frame and *L* is the number of multicast groups. In 5G communication systems, channel states are identified using a finite number of integral values termed the CQI values. The Third Generation Partnership Project (3GPP) standards [29] define a total of 15 CQI values. Since the number of CQI values is finite, the possible channel states of UEs can only take a finite number of values. Define set C that contains all possible CQI combinations of the *M* UEs in the system, i.e., for D∈N distinct CQI values, C will be a set of $D^{M}$ CQI vectors, each of length *M*. Let *g* denote the probability distribution over the set C such that, for all C∈C, the probability of the system being in the CQI state *C* is g(C).

Denote by $\mu_{B_{i}C}\in {\left\{0,1\right\}}^{M}$ the service vector of the UEs corresponding to allocation $B_{i}\in B$ in CQI state C∈C. We use $\mu_{C}={\left\{\mu_{B_{i}C}\right\}}_{B_{i}\in B}$ to denote the set of possible service vectors in channel state *C*. For a given C∈C, define a distribution $w_{C}=\left\{w_{B_{i}C}\right\}$ over the set of $\mu_{B_{i}C}$, where $w_{B_{i}C}$ denotes the probability of choosing allocation $B_{i}$ in channel state *C*.

Therefore, within this virtual queuing system, we are required to find a distribution ${\left\{w_{C}\right\}}_{C\in C}$ that satisfies the following set of constraints for δ>0:

(8a) $$\begin{array}{cc} P(\delta ): & \sum_{C\in C}\sum_{B_{i}\in B}g\left(C\right)w_{B_{i}C}\mu_{B_{i}C}=\lambda +\delta , \end{array}$$

(8b) $$\begin{array}{cc} \; & w_{B_{i}C}\geq 0,\;\forall \;B_{i}\in B,\;C\in C, \end{array}$$

(8c) $$\begin{array}{cc} \; & \sum_{B_{i}\in B}w_{B_{i}C}=1,\;\forall \;C\in C, \end{array}$$

where the constraint in (8a) ensures the stability of the virtual queuing system by imposing that the service rates of the virtual token queues are higher than their respective arrival rates, and (8b) and (8c) ensure that ${\left\{w_{C}\right\}}_{C\in C}$ is a valid probability distribution. Therefore, a policy whose assignment decisions follow the distribution ${\left\{w_{C}\right\}}_{C\in C}$ would be able to stabilize the virtual queuing system Q.

Denote by Λ(δ) the set of arrival rate vectors λ such that the feasible region of P(δ) is non-empty. We define the sets $\Lambda^{{}^{\circ}}$ and $\bar{\Lambda }$ as

(9) $$\Lambda^{{}^{\circ}}=\underset{\delta >0}{⋃}\Lambda \left(\delta \right)\;\mathrm{and}\;\bar{\Lambda }=\underset{\delta \geq 0}{⋃}\Lambda \left(\delta \right).$$

The sets $\Lambda^{{}^{\circ}}$ and $\bar{\Lambda }$ provide a means of characterizing the stability region of the constructed virtual queuing system. As we will see in the subsequent results, these sets enable us to establish the relationship between the feasible region of the optimal resource allocation policy for our system and the stability region of the constructed virtual queuing system.

The following theorem provides the relationship between $\Lambda^{{}^{\circ}}$, $\bar{\Lambda }$, and the stability region S of the queuing system Q from Definition 9.

**Theorem** **1.** *$\Lambda^{{}^{\circ}}\subseteq S\subseteq \bar{\Lambda }$.*

**Proof.**  Detailed proof is given in Appendix A. □

From here on, we consider $\Lambda^{{}^{\circ}}$ to be the stability region of Q since the region $\Lambda^{{}^{\circ}}$ is well defined for the constructed virtual queueing system. Moreover, since $\Lambda^{{}^{\circ}}\subseteq S$, all the arrival rate vectors in $\Lambda^{{}^{\circ}}$ are stabilizable.

With this result, we are now able to state the main contribution of this section, which establishes the relation between the feasible region of the optimal resource allocation policy $L^{\Gamma^{★}}$ from Definition 6 and the stability region S from Definition 9.

**Theorem** **2.** *The loss requirement of the UE is met if and only if its corresponding token queue in Q is stable. More precisely, $\tilde{\ell }\in L^{\Gamma^{★}}$ if and only if $(1-\tilde{\ell })\in S$. Here, 1 is a vector of ones of the same length as $\tilde{\ell }$.*

**Proof.**  Detailed proof is given in Appendix B. □

Theorem 2 establishes that the stability region of the virtual queuing system is the same as the feasible region of the optimal resource allocation policy $\Gamma^{★}$. Henceforth, we focus our attention on stabilizing the token queues corresponding to each UE knowing that, by Theorem 2, stabilizing the token queues of the UEs will ensure that their respective loss requirements are met.

## 5. Resource Allocation Algorithms for Loss-Tolerant Multicast Streaming

In the loss-tolerant MBMS systems under consideration, the UE is satisfied as long as the losses encountered are kept below the acceptable thresholds. In this section, we propose loss-optimal resource allocation policies that can meet the loss requirements of all UEs in the system.

### 5.1. Loss-Optimal Resource Allocation (LORA)

LORA makes scheduling decisions in a sub-frame *t* based on the token queue lengths $Q_{k}\left[t\right]$ of the users. Throughout the following, we use $\Gamma_{0}$ to denote LORA. In each sub-frame *t*, $\Gamma_{0}$ chooses a service vector $\mu^{\Gamma_{0}}\left[t\right]$ according to the following:

(10) $$\mu^{\Gamma_{0}}\left[t\right]\in \underset{\mu^{\Gamma_{0}}\left[t\right]\in \mu_{C}}{arg max}\sum_{k=1}^{M}Q_{k}\left[t\right]\mu_{k}^{\Gamma_{0}}\left[t\right],$$

where $\mu_{k}^{\Gamma_{0}}\left[t\right]$ is the service rate of UE *k* in sub-frame *t* under $\Gamma_{0}$. The intuition behind this is that $\Gamma_{0}$ maximizes the sum of the queue lengths of the UEs served in sub-frame *t*. As we have already established in Section 4, stabilizing the token queues ensures that the loss requirements of all UEs are met. Thus, in order to prove that $\Gamma_{0}$ can successfully meet the loss requirements, it is sufficient to show that $\Gamma_{0}$ stabilizes the virtual queuing system.

**Theorem** **3** (Throughput optimality of $\Gamma_{0}$)**.** *For any stabilizable arrival rate vector λ, $\Gamma_{0}$ stabilizes the queuing system.*

**Proof.**  Detailed proof is given in Appendix C. □

This theorem implies that as long as the system is stabilizable, i.e., there exists some policy Γ that can stabilize the queuing system, so can $\Gamma_{0}$. Note that Γ is not restricted to using the same information that is available to $\Gamma_{0}$. In fact, Γ could use information from the past and future allocations and channel conditions to make allocation decisions. However, the LORA policy $\Gamma_{0}$ only uses the current state of the queuing system to make the scheduling decisions.

With LORA, we now have a loss-optimal policy that meets the loss requirements of users by making allocation decisions based on the UEs’ token queue lengths. However, in addition to the amount of packet loss in a video stream, we would also like to control the pattern in which these losses occur. Even if a user has high tolerance for loss, we would like to avoid a large number of consecutive packet losses in order to improve the QoE. Starving users for a large number of consecutive sub-frames may lead to user dissatisfaction and result in users leaving the multicast session. Therefore, a loss-tolerant resource allocation policy should also restrict the number of consecutive packet losses encountered by a UE, in addition to the long-term average packet loss. We propose such a policy in the following. This policy ensures that users do not remain unserved for long periods at a time, which leads to better loss performance and the reduced burstiness of packet losses.

### 5.2. Priority LORA (p-LORA)

Similar to LORA, p-LORA also makes scheduling decisions in sub-frame *t* based on the queue lengths $Q_{k}\left[t\right]$. However, in p-LORA, we use an additional priority vector to increase the probability of serving a previously unserved UE. A similar approach has also been used in [30] to design a regular service guarantee algorithm for a wireless network. In the following, we use $\Gamma_{P}$ to denote the p-LORA policy.

In every sub-frame *t*, $\Gamma_{P}$ chooses service vector $\mu^{\Gamma_{P}}\left[t\right]$ according to the following:

(11) $$\mu^{\Gamma_{P}}\left[t\right]\in \underset{\mu^{\Gamma_{P}}\left[t\right]\in \mu_{C}}{arg max}\sum_{k=1}^{M}\left(Q_{k}\left[t\right]+\left(c_{k}\left[t\right]+1\right)\;\cdot \;s\right)\mu_{k}^{\Gamma_{P}}\left[t\right],$$

where $c_{k}\left[t\right]$ is the priority weight ascribed to the token queue of UE *k* and *s* is a positive constant. The priority weight $c_{k}\left[t\right]$ is defined as

$$c_{k}\left[t\right]=\left\{\begin{array}{cc} 0, & \mathrm{if}\;\mu_{k}\left[t-1\right]=1,\\ \min\left\{c_{k}\left[t-1\right]+1,\kappa \right\} & \mathrm{otherwise}, \end{array}\right.$$

with κ>0 being the maximum value that the priority weights can take. Additionally, for all k∈{1,2,…,M}, we set $c_{k}\left[0\right]=0$. Denote by $\bar{c}\left[t\right]=\left(c_{1}\left[t\right],\ldots ,c_{M}\left[t\right]\right)$ the vector of the priority weights of all the queues in sub-frame *t*. Increasing $c_{k}\left[t\right]$ increases the contribution of UE *k* in (11), which increases its likelihood of being served under $\Gamma_{P}$.

We now prove that $\Gamma_{P}$ is throughput-optimal, i.e., $\Gamma_{P}$ will stabilize the queuing system if any other policy can do so.

**Theorem** **4** (Throughput optimality of $\Gamma_{P}$)**.** *For any stabilizable arrival rate vector λ, $\Gamma_{P}$ stabilizes the queuing system.*

**Proof.**  Detailed proof is given in Appendix D. □

In the next section, we present the generalization of the exponential queue length (EXP-Q) rule, which was proposed in [31]. The EXP-Q rule is a well-known throughput-optimal policy for the scheduling of multiple flows over a time-varying wireless channel, such that the maximum delay encountered in the system is minimized [32]. The rule, however, considers that there is a single channel that can be used by one flow at a time. Therefore, we propose the generalization of EXP-Q for use with multicast transmission and multiple channels. It serves as a benchmark for the performance evaluation of our proposed policies.

### 5.3. Generalized Exponential (Queue Length) Rule ($\Gamma_{E}$)

The EXP-Q rule [31] schedules a single queue *k* in a time slot *t* such that

(12) $$k\in \underset{k}{arg max}\gamma_{k}\mu_{k}\left[t\right]\exp\left(\frac{a_{k}Q_{k}\left[t\right]}{\beta +{\left[\bar{Q}\left[t\right]\right]}^{\eta }}\right),$$

where $\mu_{k}\left[t\right]$ is the rate of service of queue *k* in sub-frame *t*; $a_{k}$, $\gamma_{k}$, and η are constants; and $\bar{Q}\left[t\right]=\left(1/N\right)\sum_{k}a_{k}Q_{k}\left[t\right]$. The EXP-Q rule is designed for use in a system where a single time-varying channel is shared by multiple flows. Since our considered system requires the allocation of multiple flows to multiple channels (in the form of PRBs), the EXP-Q rule cannot be used in the existing form. Therefore, we generalize it as described in the following. The generalized version of the EXP-Q rule is denoted by $\Gamma_{E}$.

Since we have multiple channels available and multiple groups can be scheduled for service in a sub-frame, the policy has to determine an allocation vector $B^{\Gamma_{E}}\left[t\right]$ instead of choosing a single entity to be scheduled in a sub-frame. As defined in Section 3, $B^{\Gamma_{E}}\left[t\right]$ is a vector that specifies which PRB is allocated to which multicast group. We define $\Gamma_{E}$ as the policy that chooses service vector $\mu^{\Gamma_{E}}\left[t\right]$ according to the following:

(13) $$\mu^{\Gamma_{E}}\left[t\right]\in \underset{\mu^{\Gamma_{E}}\left[t\right]\in \mu_{C}}{arg max}\sum_{k=1}^{M}\gamma_{k}\mu_{k}^{\Gamma_{E}}\left[t\right]\exp\left(\frac{a_{k}Q_{k}\left[t\right]}{\beta +{\left[\bar{Q}\left[t\right]\right]}^{\eta }}\right),$$

where $\mu_{k}^{\Gamma_{E}}\left[t\right]$ is the service rate of UE *k* in sub-frame *t* under $\Gamma_{E}$. Based on the service vector $\mu^{\Gamma_{E}}\left[t\right]$, the corresponding allocation vector $B^{\Gamma_{E}}\left[t\right]$ is determined.

The mapping from the service vector $\mu_{k}^{\Gamma_{E}}\left[t\right]$ to the allocation vector $B^{\Gamma_{E}}\left[t\right]$ can be accomplished as follows. Since we assume perfect CSI at the eNB, given a channel state *C* in sub-frame *t*, the eNB knows $\mu_{C}={\left\{\mu_{B_{i}C}\right\}}_{B_{i}\in B}$, the set of possible service vectors in channel state *C*. Therefore, if $\mu^{\Gamma_{E}}\left[t\right]=\mu_{B_{i}C}$, allocation vector $B^{\Gamma_{E}}\left[t\right]=B_{i}$ is chosen.

### 5.4. Computational Complexity

All the resource allocation policies discussed in this section have a brute force computational complexity of OMNLL!. This makes them unsuitable for use in practical systems unless we can find efficient means of implementing them. We show that these policies can be implemented in polynomial time using a maximum weight bipartite matching (MWBM) [33]. We discuss the details of this implementation in the next subsection.

### 5.5. Polynomial Time Implementation

We make use of MWBM for an efficient polynomial time implementation of the resource allocation policies proposed in this section. The MWBM reduces the computational complexity of their implementation to $O(NL^{2})$, where *N* is the number of PRBs in a sub-frame and *L* is the number of multicast groups. Thus, the policies can be implemented in polynomial time.

We begin with the construction of the underlying bipartite graph, which is the same for all the policies, except that the edge weights are different for each policy. In the following, we discuss the implementation for $\Gamma_{0}$ in detail. The procedure and proof can be directly used for $\Gamma_{P}$ and $\Gamma_{E}$ as well with modified edge weights. The modifications involved will be specified at the end of this section.

Construct a bipartite graph G=(U,V,E), where vertex set *U* is the set of *L* multicast groups and vertex set *V* is the set of *N* PRBs. We define the service rate of UE $k\in G_{i}$ in PRB *j* in sub-frame *t* as follows:

$$\begin{array}{c} \nu_{k}^{j}\left[t\right]=\left\{\begin{array}{cc} 0, & \mathrm{if}\;R_{i}>r_{kj}\left[t\right]\\ 1, & \mathrm{otherwise}. \end{array}\right. \end{array}$$

Note that $\mu_{k}^{\Gamma }\left[t\right]$ in (6) denotes the service rate for UE *k* under policy Γ in sub-frame *t*. Since we need to denote the service rate for a UE in each PRB here, we employ a different notation to avoid ambiguity with the service rate vector of a policy. The weight of an edge connecting i∈U to j∈V is $w_{i}^{j}\left[t\right]=\sum_{k\in G_{i}}Q_{k}\left[t\right]\nu_{k}^{j}\left[t\right]$.

In the following lemma, we show that an MWBM of G that matches each vertex in *U* to a unique vertex from *V* results in allocation equivalent to $\Gamma_{0}$.

**Lemma** **1.** *Maximum weight bipartite matching for graph G, as described above, results in resource allocation according to policy $\Gamma_{0}$.*

**Proof.**  Detailed proof is given in Appendix E. □

The same MWBM can be used to implement $\Gamma_{P}$ and $\Gamma_{E}$ by changing the edge weights. For $\Gamma_{P}$, the edge weights are given by

(14) $$w_{i}^{j}\left[t\right]=\sum_{k\in G_{i}}\left(Q_{k}\left[t\right]+\left(c_{k}\left[t\right]+1\right)\;\cdot \;s\right)\;\nu_{k}^{j}\left[t\right],$$

and, for $\Gamma_{E}$, the edge weights are

(15) $$w_{i}^{j}\left[t\right]=\sum_{k\in G_{i}}\gamma_{k}\nu_{k}^{j}\left[t\right]\exp\left(\frac{a_{k}Q_{k}\left[t\right]}{\beta +{\left[\bar{Q}\left[t\right]\right]}^{\eta }}\right).$$

In the next section, we present the numerical results of simulations performed to evaluate the performance of the proposed resource allocation schemes.

## 6. Simulations

We study the performance of the proposed allocation algorithms in an MBMS system. We consider a cell with UEs distributed uniformly at random through the cell. There are L=5 MBMS video streams available in the cell and each UE is subscribed to one of these streams. In our simulations, the same number of UEs are subscribed to each stream. All the UEs subscribed to the same video stream form a multicast group and receive the relevant content on the same PRBs. We use the MATLAB-based simulator designed in [34] for the numerical simulations. To create 5G-specific physical layer conditions, we create channels using the models recommended by 3GPP [27]. Signal-to-noise ratio (SNR) to CQI and CQI to rate mappings have been performed according to the 3GPP specifications [27]. Other relevant simulation parameters are listed in Table 2.

As described in Section 2, for each stream, a packet arrives at the beginning of a sub-frame and is transmitted in the same sub-frame at the required rate. Each UE can tolerate some amount of packet loss. We observe the packet loss encountered by the UEs under the proposed policies and compare their performance with that of the modified EXP-Q rule.

Since the proposed policies are intended for use with video streaming services, we use traces from actual videos to generate realistic video traffic patterns in the simulations. These video traces have been obtained from the Arizona State University Video Trace Library (http://trace.eas.asu.edu/ (accessed on 23 July 2019)) [4,5]. The videos used are Silence of the Lambs, Star Wars IV, the Tokyo Olympics, NBC News, and a Sony Demo. All videos are H.264/AVC, encoded with a GoP size of 16, with 15 B frames in each group.

Since I and P frames are needed to decode other frames in a GoP, we ensure that all I and P frames are transmitted without any loss by allocating sufficient resources and transmitting at the rate corresponding to the weakest UE. We use the proposed lossy allocation policies only to send the B frames. This is a recommended practice in network simulations with video traces [5], since it is difficult to estimate the impact of the loss of I and P frames on the video quality [5].

**Figure 2:**
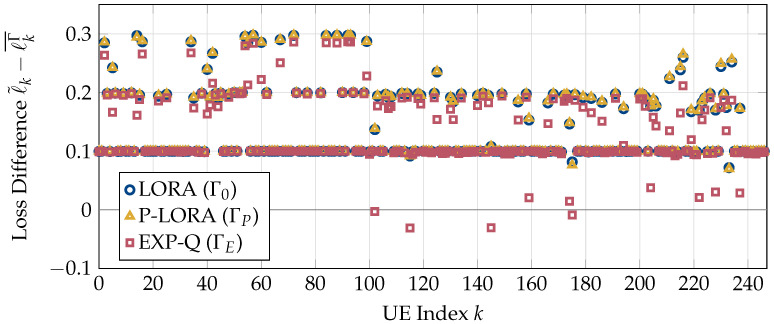
Differences between the tolerable losses ${\tilde{\ell }}_{k}$ and the average losses encountered by the video traces $\bar{\ell_{k}^{\Gamma }}$ under policies LORA, p-LORA, and EXP-Q.

First, we compare the losses encountered by the UEs to their loss tolerances. For this, we run the simulations for the entire duration of all five videos (L=5) with K=50 UEs per stream. The resulting differences between the loss tolerance ${\tilde{\ell }}_{k}$ and the average encountered loss $\bar{\ell_{k}^{\Gamma }}$ are shown in Figure 2. Note that negative values correspond to a violation of the loss tolerance. It can be seen that both LORA and p-LORA succeed in meeting the loss requirements of all UEs. In contrast, several users experience losses significantly higher than their tolerable limits for the modified EXP-Q rule.

**Figure 3:**
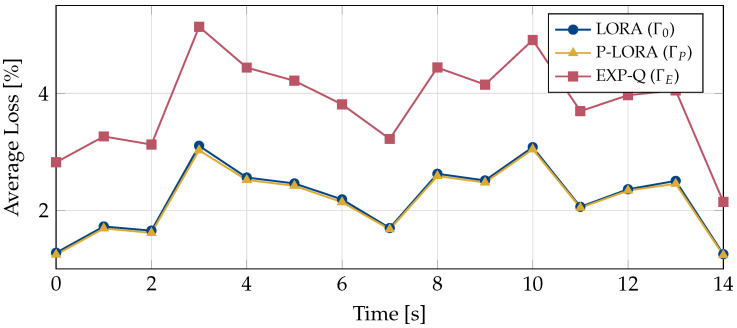
Average losses of all UEs and video streams over time for different policies.

Next, we compare the average losses encountered by the UEs under the three schemes. For this, the encountered losses per second have been exponentially averaged for each UE individually. The average of these smoothed curves over all UEs is shown in Figure 3. It can be observed that the EXP-Q rule results in the highest average loss. Both LORA and p-LORA achieve nearly the same performance, with p-LORA only being slightly better.

**Figure 4:**
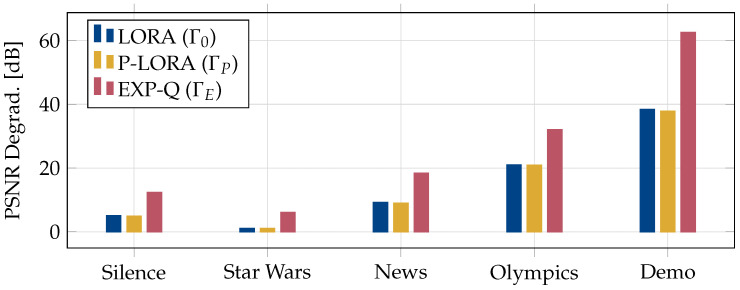
Comprarison of the PSNR degradation for different videos.

After considering the average packet loss, we compare the peak signal-to-noise ratio (PSNR) degradation due to the packet losses in Figure 4. The PSNR is widely regarded as an important metric in evaluating the quality of a video stream [5]. In order to capture the impact of each resource allocation policy on the PSNR of the transmitted videos, we plot the differences between the PSNR of the transmitted and received video streams, which we term *PSNR degradation*. This is calculated as follows.

The video traces obtained from the ASU repository contain the PSNR values of each frame in a video trace. Using these, the PSNR of a GoP is obtained by adding the PSNR values of the frames in this GoP. We then find the average PSNR of a video stream as the average over all the GoPs in this stream. This is used as the representative PSNR value for the transmitted video stream.

The PSNR of the video stream received by a UE is similarly calculated by taking the sum of the PSNR values of the frames that were successfully received by this UE within a GoP, followed by averaging over all the GoPs. We then calculate the average received PSNR of a stream by taking the average of the PSNR values over all the UEs in the group. Finally, the PSNR degradation is calculated as the difference in the average PSNR of the transmitted and received video streams. From the figure, it can be seen that EXP-Q leads to the largest degradation in the PSNR. Both LORA and p-LORA result in significantly less loss in the PSNR of the received video streams.

**Figure 5:**
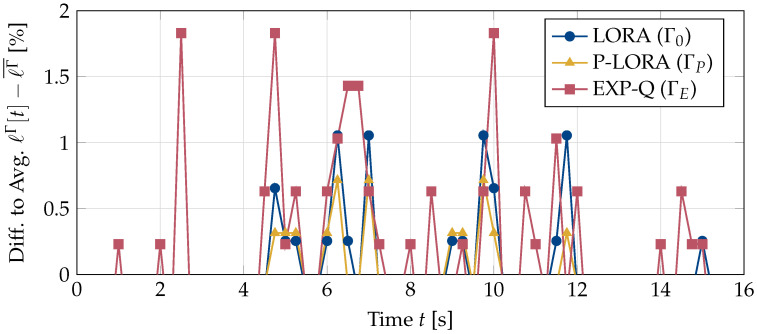
Differences between the encountered losses $\ell^{\Gamma }\left[t\right]$ for one UE at time *t* of a video stream and the average packet loss $\bar{\ell^{\Gamma }}$ for policy Γ. A high peak indicates a burst of packet loss, which can result in degradation in the video quality.

As discussed in Section 5, in addition to the amount of packet loss, the pattern in which the losses occur also has a significant impact on the user experience. While a certain amount of loss spread (more or less) evenly throughout a session may not result in a significant degradation in video quality, concentrated packet loss in a video stream can be extremely annoying and cause the UEs to leave the session. To compare the burstiness of the losses for the different policies Γ, we plot the differences between the encountered losses $\ell^{\Gamma }\left[t\right]$ in a given second *t* and the average loss $\bar{\ell^{\Gamma }}$ in Figure 5. Whenever the current loss $\ell^{\Gamma }$ is much larger than the average, a large amount of packet loss has occurred in a short period of time. As a result, the video quality is significantly worse than the average, and the users experience a degradation in the quality of service. This behavior is clearly seen for both the EXP-Q and LORA policies. On the other hand, the peaks are smaller for the p-LORA algorithm, which is specifically designed to avoid bursts of packet loss.

These simulation results clearly demonstrate the effectiveness of the proposed policies. The use of traces of actual videos further strengthens the case for the use of loss-tolerant allocation policies to stream video content.

After having shown that the proposed algorithms work effectively on real video stream data, we now focus on the influences of different parameters. In particular, we consider a varying number of video streams *L* and users per video stream *K* in the following. For the traffic data, we again use the realistic video content from the above simulations.

First, we compare the average computational time of the LORA and p-LORA algorithms in Table 3. It can be noted that the times are very similar for both algorithms. As expected, the computational effort increases both with the number of multicast groups *L* and the number of users per group *K*. However, the computational times in the simulation do not purely depend on the total number of UEs in the system LK, but also on the individual parameters. While, at L=3 and K=200, the total number of users is 600, the time of around 0.6 ms is less than the required time of around 0.8 ms for the parameters L=5 and K=100 (with a total of 500 UE). This observation that the number of video streams has a greater influence on the computational complexity matches the theoretical results derived in Section 5.5.

**Figure 6:**
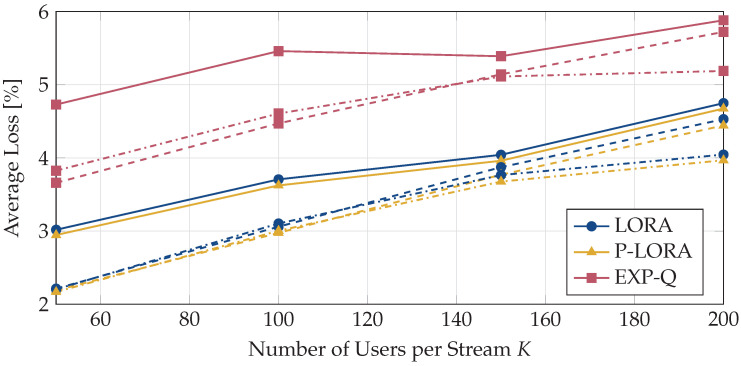
Average loss for different combinations of the number of video streams *L* and the number of users per stream *K*. The solid lines indicate the results for L=3, the dashed lines for L=4, and the dash-dotted lines for L=5.

Next, we analyze the packet losses for different combinations of *L* and *K*. In Figure 6, we show the average losses for the three algorithms LORA, p-LORA, and EXP-Q. The average is taken both with respect to the users and over time. First, it can be seen that the average loss increases for all algorithms with an increasing number of users per stream. This is because, as the number of users subscribed to a video stream increases, a larger number of users are likely to experience poor channel conditions, which in turn increases the average loss incurred by the users. For an increasing number of parallel streams, the average loss reduces. Similar to the results in Figure 3, LORA and p-LORA achieve nearly the same performance, with p-LORA being slightly better. However, both algorithms outperform the EXP-Q method.

## 7. Conclusions

Video streams can tolerate a certain amount of packet loss without a significant degradation in the quality perceived by the end user. In this paper, we have leveraged this property to improve the performance of multicast video streaming in MBMS. In particular, we have considered an MBMS system where users can tolerate a certain amount of packet loss depending on the type of video that they are streaming and the quality of their channel. For such a system, we have addressed the resource allocation problem by constructing a virtual queuing system to represent the actual loss-tolerant MBMS system. It has been shown that an optimal resource allocation policy corresponds to a policy that stabilizes the constructed virtual queuing system. Furthermore, we have proposed two algorithms, namely LORA and p-LORA, for resource allocation in loss-tolerant multicast video streaming services. Both LORA and p-LORA are optimal in the sense that their resulting resource allocation fulfills the loss requirements of all the users. Interestingly, this also implies that no policy can perform better, even if it uses information about future channel states. Additionally, we have proposed an MWBM algorithm that provides an efficient polynomial time implementation of the proposed policies. To compare our policies, we have modified the EXP-Q rule [31] for use in multicast transmission systems with multiple channels.

We have performed extensive simulations using video traces from actual video streams [5] to study and compare the performance of LORA, p-LORA, and the modified EXP-Q rule. As expected, both LORA and p-LORA are able to meet the loss requirements for all users, while EXP-Q violates this constraint for some UEs. The simulation results indicate that p-LORA achieves the smallest amount of packet loss and the best PSNR of all these policies. Therefore, we can conclude that the use of this policy to stream video content in MBMS can significantly reduce the resource utilization of video streaming services, while simultaneously satisfying the users’ video quality requirements.

## Figures and Tables

**Table 1:** Notation of the most commonly used variables.

Symbol	Explanation	Definition
Γ	Resource allocation policy	Definition 1
$B^{\Gamma }\left[t\right]$	Allocation vector for sub-frame *t*	Equation (1)
$\ell_{k}^{\Gamma }\left[t\right]$	Packet loss of UE *k* in sub-frame *t*	Equation (2)
${\tilde{\ell }}_{k}$	Tolerated fractional loss of UE *k*	—
$\bar{\ell_{k}^{\Gamma }}$	Average packet loss of UE *k*	Definition 3
$\mu_{k}^{\Gamma }\left[t\right]$	Service indicator for UE *k* in sub-frame *t*	Equation (6)
${\bar{\lambda }}_{k}$	Average arrival rate of virtual queue *k*	${\bar{\lambda }}_{k}=1-{\tilde{\ell }}_{k}$
$Q_{k}^{\Gamma }\left[t\right]$	Queue length of UE *k* in sub-frame *t*	Equation (7)

**Table 2:** System simulation parameters [27].

Parameter	Value
System bandwidth	20MHz
Path loss model	$128.1+37.6\log_{10}(d)$, with *d* in km
Lognormal shadowing	Log-normal fading with 10dB standard deviation
White noise power density	−174dBm/Hz
eNB cell radius	150 m
eNB noise figure	5dB
eNB transmit power	46dBm
Number of PRBs	100 per sub-frame

**Table 3:** Average time taken for resource allocation using the MWBM implementation of the LORA and p-LORA algorithms. Each row represents a different number of video streams *L* and each column represents different number of UEs *K* subscribed to each stream. The first number indicates the time required to run the LORA algorithm, while the number in parentheses is for p-LORA.

*L*	K=50	K=100	K=150	K=200
3	0.439 (0.438) ms	0.495 (0.497) ms	0.546 (0.543) ms	0.619 (0.613) ms
4	0.571 (0.571) ms	0.663 (0.660) ms	0.723 (0.721) ms	0.830 (0.825) ms
5	0.697 (0.706) ms	0.819 (0.813) ms	0.905 (0.894) ms	1.000 (1.000) ms

## Data Availability

The video traces used for the simulations in Section 6 are part of the Arizona State University Video Trace Library (http://trace.eas.asu.edu/) [5].

## Abbreviations

The following abbreviations are used in this paper:

3GPP	Third Generation Partnership Project
CQI	channel quality indicator
DTMC	discrete time Markov chain
EXP-Q	exponential queue length
FDPS	frequency domain packet scheduler
GoP	group of pictures
LORA	loss-optimal resource allocation
MBMS	multimedia broadcast multicast services
MWBM	maximum weight bipartite matching
NOMA	non-orthogonal multiple access
p-LORA	priority LORA
PRB	physical resource block
PSNR	peak signal-to-noise ratio
QoE	quality of experience
QoS	quality of service
SLLN	strong law of large numbers
SNR	signal-to-noise ratio
UE	user equipment

## Appendix A. Proof of Theorem 1

We begin by constructing the following randomized resource allocation policy $\Gamma_{\delta }$ based on the set of constraints P(δ) in (8).

**Definition** **A1** (Randomized allocation policy $\Gamma_{\delta }$)**.** *$\Gamma_{\delta }$ chooses an allocation vector in a sub-frame according to a feasible solution $w_{C}$ of P(δ). If the system is in channel state C, $\Gamma_{\delta }$ chooses allocation vector $B_{i}$ with probability $w_{B_{i}C}$, i.e., $\mathrm{Pr}\left(B^{\Gamma_{\delta }}\left[t\right]=B_{i}\;|\;C\left(t\right)=C\right)=w_{B_{i}C},\forall \;t$, and decisions across sub-frames are independent. δ is an input parameter for $\Gamma_{\delta }$.*

The definition of $\Gamma_{\delta }$ will be used to prove various results in this and the following sections. Consider the arrival rate vector $\lambda ={\left({\bar{\lambda }}_{1},\ldots ,{\bar{\lambda }}_{M}\right)}^{T}\in \Lambda^{{}^{\circ}}$. By the definition of $\Lambda^{{}^{\circ}}$ in (9), there exists δ>0 such that P(δ) is feasible for arrival rate vector λ. Let $w_{C}=\left\{w_{B_{i}C}\right\}$ denote a feasible solution of P(δ). Therefore, we can use policy $\Gamma_{\delta }$ to make scheduling decisions in each sub-frame according to $w_{C}$. Let $A_{k}\left[t\right]$ denote the arrival process of queue *k*, with $A_{k}\left[t\right]=1$ if there is an arrival to queue *k* in sub-frame *t* and 0 otherwise. $D_{k}^{\Gamma_{\delta }}\left[t\right]$ denotes the departure process of *k* under $\Gamma_{\delta }$. We have $D_{k}^{\Gamma_{\delta }}\left[t\right]=1$ if a token departs from *k* under $\Gamma_{\delta }$ in sub-frame *t* and 0 otherwise. Therefore, the evolution of the queue length of queue *k* under $\Gamma_{\delta }$ is given by

(A1) $$Q_{k}^{\Gamma_{\delta }}\left[t+1\right]={\left[Q_{k}^{\Gamma_{\delta }}\left[t\right]+A_{k}\left[t\right]-D_{k}^{\Gamma_{\delta }}\left[t\right]\right]}^{+},$$

where $Q_{k}^{\Gamma_{\delta }}\left[t\right]$ is the length of the token queue of UE *k* at time *t* under policy $\Gamma_{\delta }$. For simplicity of notation, we omit the $\Gamma_{\delta }$ superscript from $Q_{k}^{\Gamma_{\delta }}\left[t\right]$ and $D_{k}^{\Gamma_{\delta }}\left[t\right]$ throughout the rest of this section. Since a departure from queue *k* means that UE *k* was successfully served, the corresponding service rate is $\mu_{k}^{\Gamma_{\delta }}\left[t\right]=1$, and we can write (A1) as follows.

(A2) $$Q_{k}\left[t+1\right]={\left[Q_{k}\left[t\right]+A_{k}\left[t\right]-\mu_{k}^{\Gamma_{\delta }}\left[t\right]\right]}^{+}.$$

The state of the queuing system in a sub-frame can be completely defined by the queue lengths of all the token queues in the sub-frame. We denote the state of the system in sub-frame *t* by the vector $Q[t]=[Q_{1}\left[t\right],\ldots ,Q_{M}\left[t\right]]$. Since the scheduling decisions made under $\Gamma_{\delta }$ only make use of the current state of the system, the evolution of the states of the system ${\left\{Q\left[t\right]\right\}}_{t\geq 0}$ under $\Gamma_{\delta }$ forms a discrete time Markov chain (DTMC). This DTMC is countable, irreducible, and aperiodic. We prove this in the following result.

**Lemma** **A1.**
*The DTMC ${\left\{Q\left[t\right]\right\}}_{t\geq 0}$ formed by the evolution of the states of the system under policy $\Gamma_{\delta }$ is countable, irreducible, and aperiodic.*

**Proof.** *Countable:* The state space of the DTMC is the set of all *M*-tuples $\left(Q_{1}\left[t\right],\ldots ,Q_{M}\left[t\right]\right)$, where $Q_{k}\left[t\right]\in N$. It forms an *M*-dimensional Cartesian product of the set of natural numbers N, which is a countable set. Therefore, the state space of the DTMC and hence the DTMC itself is countable [35] [Thm. 2.13]. *Irreducible*: The DTMC can transition from any state Q to a state $Q^{\prime }$ in the following steps:

1. Schedule all UEs for service until all queues are empty. This is accomplished in $\max_{k}Q_{k}$ sub-frames.
2. For the next $\max_{k}Q_{k}^{\prime }$ sub-frames, the token queue of UE *k* has an arrival and no departure for the first $Q_{k}^{\prime }$ sub-frames. In the remaining $\left(\max_{k}Q_{k}^{\prime }-Q_{k}^{\prime }\right)$ sub-frames, there is no new arrival and no departure. At the end of this step, the DTMC is in state $Q^{\prime }$.

These steps define at least one path of length ($\max_{k}Q_{k}+\max_{k}Q_{k}^{\prime }$) from any state Q to any other state $Q^{\prime }$. Therefore, the DTMC is irreducible. *Aperiodic*: If the DTMC is in state Q[t] and no new token arrives in any queue and no queue is scheduled for service, the state of the DTMC remains unchanged. Therefore, self-loops exist and the DTMC is aperiodic. □

We now begin the proof of Theorem 1. This is done in two steps. First, we establish in Lemma A2 that $\Lambda^{{}^{\circ}}\subseteq S$, and we then show that $S\subseteq \bar{\Lambda }$ in Lemma A3.

**Lemma** **A2.**
*Every $\lambda \in \Lambda^{{}^{\circ}}$ is a stabilizable arrival rate vector. Hence, $\Lambda^{{}^{\circ}}\subseteq S$.*

**Proof.** To prove this, we first show, using Foster’s theorem [36], that the DTMC ${\left\{Q\left[t\right]\right\}}_{t\geq 0}$ is positive recurrent and hence the queue lengths do not grow infinitely under $\Gamma_{\delta }$. Using the Lyapunov function $f\left(Q[t]\right)=\sum_{k=1}^{M}Q_{k}^{2}\left[t\right]$, we have

$$\begin{array}{c} f(Q[t+1])-f(Q[t])\leq \sum_{k=1}^{M}\left[{\left(A_{k}\left(t\right)-\mu_{k}^{\Gamma_{\delta }}\left[t\right]\right)}^{2}+2Q_{k}\left[t\right]\left(A_{k}\left[t\right]-\mu_{k}^{\Gamma_{\delta }}\left[t\right]\right)\right]. \end{array}$$

Hence,

$$\begin{array}{cc} E\left[f(Q[t+1])-f(Q[t])\;|\;Q[t]\right]\; & \leq E\left[\sum_{k=1}^{M}{\left(\;A_{k}\left(t\right)-\mu_{k}^{\Gamma_{\delta }}\left[t\right]\right)}^{2}+2Q_{k}\left[t\right]\left(\;A_{k}\left[t\right]-\mu_{k}^{\Gamma_{\delta }}\left[t\right]\right)|Q[t]\right]\\ \; & \leq M+2E\left[\sum_{k=1}^{M}Q_{k}\left[t\right]A_{k}\left[t\right]-\sum_{k=1}^{M}Q_{k}\left[t\right]\mu_{k}^{\Gamma_{\delta }}\left[t\right]\;|\;Q[t]\right]\\ \; & \leq M+2\sum_{k=1}^{M}Q_{k}\left[t\right]{\bar{\lambda }}_{k}-2\sum_{k=1}^{M}Q_{k}\left[t\right]E\left[\mu_{k}^{\Gamma_{\delta }}\left[t\right]\;|\;Q[t]\right]. \end{array}$$

From P(δ), we have $E\left[\mu_{k}^{\Gamma_{\delta }}\left[t\right]\;|\;Q[t]\right]={\bar{\lambda }}_{k}+\delta$. Therefore,

$$\begin{array}{cc} E\left[f(Q[t+1])-f(Q[t])\;|\;Q[t]\right]\; & \leq M+2\sum_{k=1}^{M}Q_{k}\left[t\right]{\bar{\lambda }}_{k}-2\sum_{k=1}^{M}Q_{k}\left[t\right]\left({\bar{\lambda }}_{k}+\delta \right)\\ \; & \leq M-2\sum_{k=1}^{M}Q_{k}\left[t\right]\delta . \end{array}$$

Defining set $A=\left\{Q:\sum_{k=1}^{M}Q_{k}\leq \frac{M+1}{2\delta }\right\}$, we have

$$\begin{array}{c} E\left[f(Q[t+1])-f(Q[t])\;|\;Q[t]\right]<\left\{\begin{array}{cc} -1, & \forall \;Q[t]\notin A,\\ \infty , & \mathrm{otherwise}. \end{array}\right. \end{array}$$

Thus, by Foster’s theorem [36], the DTMC is positive recurrent, so the expected queue lengths in the queuing system are finite. Therefore, $\Gamma_{\delta }$ stabilizes the system for arrival rate vector $\lambda \in \Lambda^{{}^{\circ}}$. Thus, λ∈S, which implies that $\Lambda^{{}^{\circ}}\subseteq S$. □

This proves the first part of our result. We now need to show that $S\subseteq \bar{\Lambda }$. In the interest of simplicity of notation, we assume that, under a policy Γ that stabilizes the system, the following limit exists with probability 1:

(A3) $$\lim_{T\to \infty }\frac{1}{T}\sum_{t=1}^{T}1_{B_{i}C}^{\Gamma }\left[t\right],$$

where $1_{B_{i}C}^{\Gamma }\left[t\right]$ is an indicator random variable that is 1 if allocation vector $B_{i}$ is chosen by Γ under channel state *C* in sub-frame *t* and zero otherwise. Now, consider the following sets of sample paths.

A1: The set of sample paths on which the strong law of large numbers (SLLN) holds for the arrival rates, i.e., $\frac{\sum_{i=1}^{t}\lambda_{k}\left[i\right]}{t}\to {\bar{\lambda }}_{k}$ as t→∞,∀k. This is a probability 1 set, i.e., $\mathrm{Pr}(A_{1})=1$.
A2: The set of sample paths on which $\frac{\sum_{i=1}^{t}1_{\left\{C(i)=C\right\}}}{t}\to g\left(C\right)$ as t→∞,∀C (SLLN holds), where $1_{\left\{C(t)=C\right\}}$ is an indicator random variable that is 1 if the channel state in sub-frame *t* is *C* and 0 otherwise. Since *g* is a probability distribution over the set of channel states C, we have $\mathrm{Pr}(A_{2})=1$.
A3: The set of sample paths on which the service rate under Γ is greater than or equal to λ. Since Γ stabilizes the system, we have $\mathrm{Pr}(A_{3})=1$.
A4: The set of sample paths over which the limit in (A3) exists. Since we assume that this limit exists with probability 1, $\mathrm{Pr}(A_{4})=1$.

Since $A_{1},A_{2},A_{3},A_{4}$ are probability 1 sets, their intersection, $A={⋂}_{i=1}^{4}A_{i}$, is also a probability 1 set. We refer to the sample paths belonging to this set *A* as *non-trivial sample paths*. We now prove the second part of our result.

**Lemma** **A3.**
*If $\lambda \notin \bar{\Lambda }$, then λ∉S. Thus, $S\subseteq \bar{\Lambda }$.*

**Proof.** We prove this result using a contradiction. Let $\lambda \notin \bar{\Lambda }$ be a stabilizable arrival rate vector, i.e., λ∈S. Since λ is a stabilizable arrival rate vector, there exists some allocation policy Γ that can stabilize the system for arrival rate λ. We observe the scheduling decisions made by this Γ along a non-trivial sample path from the set $A={⋂}_{i=1}^{4}A_{i}$. Let $v_{B_{i}C}$ denote the fraction of time for which Γ chooses the allocation vector $B_{i}$ in channel state *C* along such a sample path. Since Γ stabilizes the system, the rate of departures must equal the arrival rate in the system. Therefore, it follows that

(A4) $$\begin{array}{cc} \; & \sum_{C\in C}\sum_{B_{i}\in B}g\left(C\right)v_{B_{i}C}\mu_{B_{i}C}=\lambda , \end{array}$$

(A5) $$\begin{array}{cc} \mathrm{where},\; & v_{B_{i}C}\geq 0\;\forall \;B_{i}\in B,\;C\in C, \end{array}$$

(A6) $$\begin{array}{cc} \; & \sum_{B_{i}\in B}v_{B_{i}C}=1\;\forall \;C\in C. \end{array}$$

This implies that $v=\{v_{B_{i}C}\}$ is a feasible solution of P(δ) and that

(A7) $$\lambda \in \Lambda \left(0\right)\Rightarrow \lambda \in \bar{\Lambda },$$

which is a contradiction. Therefore, $\lambda \notin \bar{\Lambda }$ is not stabilizable, i.e., any stabilizable λ must be contained in $\bar{\Lambda }$. Hence, we have that $S\subseteq \bar{\Lambda }$. □

From Lemmas A2 and A3, we have $\Lambda^{{}^{\circ}}\subseteq S\subseteq \bar{\Lambda }$, which is the required result.

## Appendix B. Proof of Theorem 2

We need to show that the loss requirement of a UE is met if and only if its token queue in the queuing system is stable. First, we argue that the stability of the queuing system implies that the loss requirements of the UEs are met. If the queue corresponding to UE *k* is stable, it means that there exists a policy Γ that stabilizes the queue for $\lambda \in \Lambda^{{}^{\circ}}$. We can, therefore, construct a randomized policy $\Gamma_{\delta }$ as defined in Definition A1 above. Under $\Gamma_{\delta }$, the rate of service is greater than ${\bar{\lambda }}_{k}$, which means that UE *k* is served in more than $\left(1-{\tilde{\ell }}_{k}\right)$ of the sub-frames. Therefore, the loss encountered by UE *k* is less than ${\tilde{\ell }}_{k}$ and its loss requirement is met.

Now, let us assume that the loss requirement of UE *k* is met. We show that this ensures the stability of its token queue. Since the loss requirement ${\tilde{\ell }}_{k}$ is achievable, there exists a policy Γ that satisfies the loss requirement. This means that, under Γ, the UE is being served in more than $\left(1-{\tilde{\ell }}_{k}\right)$ of the sub-frames. Since the arrival rate ${\bar{\lambda }}_{k}=\left(1-{\tilde{\ell }}_{k}\right)$, the queue is served at a rate greater than the arrival rate. Hence, Γ stabilizes the token queue. From these arguments, we conclude that the loss requirement of a UE is met if and only if its corresponding token queue in the queuing system is stable. Therefore, the feasible region of the optimal allocation policy $\Gamma^{★}$, $L^{\Gamma^{★}}$, is equivalent to the stability region of the queuing system S.

## Appendix C. Proof of Theorem 3

Similar to (A2) in Appendix A, the evolution of the queue length in the token queue of UE *k* at time *t* under $\Gamma_{0}$ is given by $Q_{k}\left[t+1\right]={\left[Q_{k}\left[t\right]+A_{k}\left[t\right]-\mu_{k}^{\Gamma_{0}}\left[t\right]\right]}^{+}$. The state of the queuing system is completely defined by the vector $Q[t]=[Q_{1}\left[t\right],\ldots ,Q_{M}\left[t\right]]$. The evolution of Q[t] forms a DTMC since the scheduling decisions made by $\Gamma_{0}$ in a sub-frame are based solely on the state of the system in the sub-frame. The DTMC ${\left\{Q\left[t\right]\right\}}_{t\geq 0}$ is countable, irreducible, and aperiodic. This can be easily proven following the same arguments as in the proof of Lemma A1 in Appendix A. We now show, using Foster’s theorem [36], that this DTMC is positive recurrent and hence the token queues do not grow infinitely.

Using the same Lyapunov function *f* and similar arguments as in the proof of Lemma A2 in Appendix A, it follows that

(A8) $$\begin{array}{cc} E\left[f(Q[t+1])-f(Q[t])\;|\;Q[t]\right]\; & =E\left[\sum_{k=1}^{M}\;{\left(\;A_{k}\left(t\right)-\mu_{k}^{\Gamma_{0}}\left[t\right]\right)}^{2}+2Q_{k}\left[t\right]\left(\;A_{k}\left[t\right]-\mu_{k}^{\Gamma_{0}}\left[t\right]\right)|Q[t]\right]\\ \; & \leq M+2\sum_{k=1}^{M}Q_{k}\left[t\right]{\bar{\lambda }}_{k}-2E\left[\sum_{k=1}^{M}Q_{k}\left[t\right]\mu_{k}^{\Gamma_{0}}\left[t\right]|Q[t]\right]. \end{array}$$

Let $\mu_{k}^{\Gamma_{\delta }}\left[t\right]$ denote the service rate for UE *k* in sub-frame *t* under the randomized policy $\Gamma_{\delta }$. Then, from (10), we have

(A9) $$\sum_{k=1}^{M}Q_{k}\left[t\right]\mu_{k}^{\Gamma_{0}}\left[t\right]\geq \sum_{k=1}^{M}Q_{k}\left[t\right]\mu_{k}^{\Gamma_{\delta }}\left[t\right].$$

Therefore, from (A8) and (A9),

$$\begin{array}{cc} E\left[f(Q[t+1])-f(Q[t])\;|\;Q[t]\right]\; & \leq M+2\sum_{k=1}^{M}Q_{k}\left[t\right]{\bar{\lambda }}_{k}-2E\left[\sum_{k=1}^{M}Q_{k}\left[t\right]\mu_{k}^{\Gamma_{\delta }}\left[t\right]\;|\;Q[t]\right]\\ \; & \leq M+2\sum_{k=1}^{M}Q_{k}\left[t\right]{\bar{\lambda }}_{k}-2\sum_{k=1}^{M}Q_{k}\left[t\right]\left({\bar{\lambda }}_{k}+\delta \right)\\ \; & \leq M-2\sum_{k=1}^{M}Q_{k}\left[t\right]\delta . \end{array}$$

Defining set $A=\{Q:\sum_{k=1}^{M}Q_{k}\leq \frac{M+1}{2\delta }\}$ and following the same arguments as in the proof of Lemma A2, it follows that the DTMC is positive recurrent. Therefore, $\Gamma_{0}$ stabilizes the system.

## Appendix D. Proof of Theorem 4

When using policy $\Gamma_{P}$ for resource allocation, the state of the queuing system is completely defined by the queue length and the value of the priority counter of each queue. We denote the state of the queuing system in sub-frame *t* under policy $\Gamma_{P}$ by $Q^{\Gamma_{P}}\left[t\right]=\left(Q_{1}^{\Gamma_{P}}\left[t\right],\ldots ,Q_{M}^{\Gamma_{P}}\left[t\right],\bar{c}\left[t\right]\right)$. Since scheduling decisions under $\Gamma_{P}$ in a sub-frame are based only on the state of the system in the sub-frame, the evolution of the states of the system form a DTMC that is countable, irreducible, and aperiodic. This can be easily proven following similar arguments as in Lemma A1 in Appendix A. We omit the details here in the interest of space. We now proceed to prove Theorem 4 as follows.

Similar to (A2) in Appendix A, the evolution of the queue length in the token queue of UE *k* at time *t* under $\Gamma_{P}$ is given by $Q_{k}\left[t+1\right]={\left[Q_{k}\left[t\right]+A_{k}\left[t\right]-\mu_{k}^{\Gamma_{P}}\left[t\right]\right]}^{+}$. The evolution of the state of the queuing system $Q[t]=[Q_{1}\left[t\right],\ldots ,Q_{M}\left[t\right],\bar{c}\left[t\right]]$ forms a DTMC that is countable, irreducible, and aperiodic. We now show, using Foster’s theorem [36], that this DTMC is positive recurrent and hence the queues do not grow infinitely.

Using the same Lyapunov function *f* and following similar steps as in the proof of Theorem 3 in Appendix C, we have

(A10) $$E\left[f(Q[t+1])-f(Q[t])\;|\;Q[t]\right]\leq M+2\sum_{k=1}^{M}Q_{k}\left[t\right]{\bar{\lambda }}_{k}-2E\left[\sum_{k=1}^{M}Q_{k}\left[t\right]\mu_{k}^{\Gamma_{P}}\left[t\right]\;|\;Q[t]\right].$$

Let $\mu_{k}^{\Gamma_{\delta }}\left[t\right]$ denote the service rate for UE *k* in sub-frame *t* under the randomized policy $\Gamma_{\delta }$. Then, from (11), we have

(A11) $$\sum_{k=1}^{M}\left(Q_{k}\left[t\right]\mu_{k}^{\Gamma_{P}}\left[t\right]+\left(c_{k}\left[t\right]+1\right)s\mu_{k}^{\Gamma_{P}}\left[t\right]\right)\geq \sum_{k=1}^{M}\left(Q_{k}\left[t\right]\mu_{k}^{\Gamma_{\delta }}\left[t\right]+\left(c_{k}\left[t\right]+1\right)s\mu_{k}^{\Gamma_{\delta }}\left[t\right]\right).$$

Therefore, from (A10) and (A11),

$$\begin{array}{cc} \; & E\left[f(Q[t+1])-f(Q[t])\;|\;Q[t]\right]\\ \; & \leq M+2\sum_{k=1}^{M}Q_{k}\left[t\right]{\bar{\lambda }}_{k}-2E\left[\sum_{k=1}^{M}Q_{k}\left[t\right]\mu_{k}^{\Gamma_{\delta }}\left[t\right]+\left(c_{k}\left[t\right]+1\right)\left(\mu_{k}^{\Gamma_{\delta }}\left[t\right]-\mu_{k}^{\Gamma_{P}}\left[t\right]\right)s|Q[t]\right]\\ \; & \leq M-2\sum_{k=1}^{M}Q_{k}\left[t\right]\delta +4Ms.\;\left(\mathrm{for}\;\kappa =1\right) \end{array}$$

Defining set $A=\left\{Q:\sum_{k=1}^{M}Q_{k}\leq \frac{4Ms+M+1}{2\delta }\right\}$, and following the same arguments as in the proof of Lemma A2, it follows that the DTMC is positive recurrent. Thus, $\Gamma_{P}$ stabilizes the system.

## Appendix E. Proof of Lemma 1

The matching for graph G selects edges that share no common vertices. This means that each group from *U* will be matched to exactly one PRB from *V* and each PRB from *V* will be matched to, at most, one group from *U*. Therefore, the requirement of assigning no more than 1 PRB to each group is satisfied. Since PRBs in *V* are matched to no more than one group from *U*, we will have $B_{i}^{\Gamma }\left[t\right]\neq B_{i^{\prime }}^{\Gamma }\left[t\right]\;\forall \;\left\{i,i^{\prime }\in \left[L\right]:B_{i}^{\Gamma }\left[t\right],B_{i^{\prime }}^{\Gamma }\left[t\right]\neq 0\right\}$, as required by Definition 1. Thus, the solution of the MWBM provides feasible resource allocation. Next, we show that the resulting allocation is consistent with the allocation decisions that would be made by policy $\Gamma_{0}$.

MWBM selects edges such that the sum of the weights of the edges chosen is maximized. Therefore, it maximizes the quantity $\sum_{i\in U}\sum_{k\in G_{i}}Q_{k}\left[t\right]\nu_{k}^{j}\left[t\right]=\sum_{k=1}^{M}Q_{k}\left[t\right]\mu_{k}^{\Gamma_{0}}\left[t\right]$, which is the same as in (10). Hence, the resource allocation performed using MWBM on G is consistent with policy $\Gamma_{0}$.
